# Insulin Resistance in Obese Children and Adolescents: HOMA−IR Cut−Off Levels in the Prepubertal and Pubertal Periods

**DOI:** 10.4274/jcrpe.v2i3.100

**Published:** 2010-08-02

**Authors:** Selim Kurtoğlu, Nihal Hatipoğlu, Mümtaz Mazıcıoğlu, Mustafa Kendirici, Mehmet Keskin, Meda Kondolot

**Affiliations:** 1 Erciyes University Faculty of Medicine, Department of Pediatric Endocrinology, Kayseri, Turkey; 2 Erciyes University Faculty of Medicine, Department of Family Medicine, Kayseri, Turkey; 3 Gaziantep University Faculty of Medicine, Department of Pediatric Endocrinology, Gaziantep, Turkey; 4 Erciyes University Faculty of Medicine, Department of Pediatrics, Unit of Social Pediatrics, Kayseri, Turkey; +90 352 437 52 85+90 536 323 03 02nihalhatipoglu@yahoo.comDepartment of Pediatric Endocrinology, Erciyes University Faculty of Medicine, Kayseri, Turkey

**Keywords:** obesity, insulin resistance, pubertal status, gender, HOMA−IR cut−off values

## Abstract

**Objective**: Childhood obesity is associated with an increased risk for insulin resistance. The underlying mechanism for the physiological increase in insulin levels in puberty is not clearly understood. The aim of the present study was to determine the cut−off values for homeostasis model assessment for insulin resistance (HOMA−IR) in obese children and adolescents according to gender and pubertal status.

**Methods**: Two hundred and eight obese children and adolescents (141 girls, 127 boys) aged between 5 and 18 years were included in the study. The children were divided into prepubertal and pubertal groups. A standard oral glucose tolerance test (OGTT) was carried out in all children. A total insulin level exceeding 300 μU/mL in the blood samples, collected during the test period, was taken as the insulin resistance criterion. Cut−off values for HOMA−IR were calculated by receiver operating characteristic (ROC) analysis.

**Results**: In the prepubertal period, the rate of insulin resistance was found to be 37% in boys and 27.8% in girls,while in the pubertal period, this rate was 61.7% in boys and 66.7% in girls. HOMA−IR cut−off values for insulin resistance in the prepubertal period were calculated to be 2.67 (sensitivity 88.2%, specificity 65.5%) in boys and 2.22 (sensitivity 100%, specificity 42.3%) in girls, and in the pubertal period, they were 5.22 (sensitivity 56%, specificity 93.3%) in boys and 3.82 (sensitivity 77.1%, specificity 71.4%) in girls.

**Conclusions**: Since gender, obesity and pubertal status are factors affecting insulin resistance, cut−off values which depend on gender and pubertal status, should be used in evaluation of insulin resistance.

**Conflict of interest:**None declared.

## INTRODUCTION

Insulin resistance and glucose intolerance are frequent in obese children and adolescents and lead to a significant risk for hypertension and cardiovascular diseases, as well as for type 2 diabetes (1, 2, 3, 4). The development of type 2 diabetes mellitus is induced by the decreased insulin sensitivity, which leads to increased insulin production. This imbalance causes a predisposition to several metabolic disorders such as early atherosclerosis, progressive obesity, canthosis nigricans, increase in skin tags, hypertension, dyslipidemia, fatty liver and polycystic ovarian syndrome (5). Obesity and insulin resistance, previously considered as a problem of older ages, are becoming serious issue also in the pediatric age group. Early detection of insulin resistance is important for prevention of these complications.

Various methods to define insulin sensitivity have been developed. Among other models, euglycemic clamp and modified minimal model are considered to be gold standards. However, they are complex and invasive tests, which can be used for research purposes only (6, 7). Oral glucose tolerance test (OGTT) has been shown to be as reliable as a frequently sampled intravenous glucose tolerance test (FSIVGTT ) and a clamp test in determining insulin sensitivity (8, 9). However, the use of OGTT in large populations is limited. Therefore, methods such as fasting insulin level, fasting glucose/insulin ratio (FGIR), homeostasis model assessment for insulin resistance (HOMA−IR) and quantitative insulin−sensitivity check index (QUICKI) are frequently used in population screening (7, 10, 11, 12). HOMA−IR was found to be much more reliable than FGIR and QUICKI in determining insulin resistance in obese children (13). HOMA−IR is a frequently used parameter in clinical research (12, 14).

Studies have shown that a physiological transient insulin resistance develops in the pubertal period although its cause is not fully understood. Despite the fact that HOMA−IR cut−off limit for insulin resistance is accepted as 2.5 in adults, corresponding values in prepubertal and pubertal children and adolescents have not been reported (10). It is known that the frequency of insulin resistance varies between genders and also among races (15, 16, 17, 18, 19, 20). However, to our knowledge, there are no studies defining cut−off levels for HOMA−IR in children of prepubertal and pubertal ages (14, 21, 22, 23).

The aim of the present study was to determine HOMA−IR cut−off values in obese children and adolescents according to gender and pubertal status.

## METHODS

Two hundred and sixty eight obese children and adolescents (141 girls, 127 boys) aged between 5 and 18 years were included in the study. All patients had presented with obesity to the Pediatric Endocrinology Clinic at Erciyes University, Faculty of Medicine. Those with an underlying endocrinologic disease or/and those under medication were excluded from the study. The children included in the study had normal thyroid function tests and morning fasting cortisol levels. Ethical approval was obtained from the Institutional Review Board of Erciyes University, Faculty of Medicine. Parental consent was taken for additional blood sampling to determine insulin levels. Body mass index (BMI) of the patients was calculated using the equation: BMI = Body weight (Kg)/Height (m)^2^. Subjects, whose BMI values were above the 95^th^ percentile of the BMI reference curve adjusted to Turkish children according to age and gender, were accepted as obese (24). Detailed history was taken from the parents, and the subjects underwent a detailed physical examination and pubertal evaluation performed by the same pediatric endocrinologist. A testicular volume equal to or greater than 4 ml in boys and onset of breast development in girls were accepted as the criteria for onset of puberty.

OGTT was carried out in order to determine insulin resistance. Following a 3−day high carbohydrate diet (300 g /day) and overnight fasting, an oral dose of 1.75 g/kg (maximum 75 g) glucose was given, and blood samples were taken at 0, 30, 60, 90 and 120 minutes from a venous catheter for glucose and insulin assessments (25, 26). A total (the sum of insulin levels at 0, 30, 60, 90, and 120 minutes during the OGTT) insulin level exceeding 300 μU/mL was taken as hyperinsulinemia. This method was first introduced by Maruhama at al (25). The main reason for selecting this criterion for hyperinsulinemia was to determine a cut−off for HOMA−IR as an index of insulin resistance. At each point in OGTT, both glucose and insulin levels were measured, and then, total insulin levels exceeding 300 μU/mL were recorded as hyperinsulinemia. Glucose levels at 120th minute were taken as a criterion for impaired glucose tolerance or diabetes mellitus. HOMA−IR was calculated using the equation: HOMA−IR=Fasting insulin (μU/mL) x Fasting glucose (mg/dL) /405 (10).

**Biochemical Analysis**

Plasma glucose levels were measured by the glucose oxidase method and the modified Trinder color reaction catalyzed by the peroxidase enzyme. Insulin levels were measured with a immunoradiometric assay kit (INS−Irma Biosource, Nivelles, Belgium). The assay detection limit was 1 μU/mL, and intra− and interassay coefficients of variation were 2.2% and 6.5%, respectively.

**Statistical Analysis**

All statistical analyses were carried out using the Statistical Package for Social Sciences (SPSS version 15.0 for Windows, Chicago, IL) and MedCalc (version 7.4.1.1, written by Frank Schoonjans, Mariakerke, Belgium). For the diagnosis of insulin resistance, HOMA−IR values were obtained with the receiver operating characteristic (ROC) analysis. Total insulin values exceeding 300 μU/mL during the OGTT were accepted as insulin resistance, and ROC analysis was undertaken to determine HOMA−IR cut−off values accordingly. ROC analysis is used to assess the actual correctness of research results. Among the terms used in this analysis, sensitivity is the ratio of patients with positive results (TP) to the number of patients, while specificity is the ratio of healthy individuals with negative results (TN) to the number of healthy individuals (27). ROC analysis is a standard approach used for determining the sensitivity and specificity of the diagnostic procedure. ROC curves to find out the relationship between the sensitivity and specificity of the diagnosis are used for this purpose. The curves are between the limits 0 and 1, and while proximity to the y coordinate and the upper limit shows a successful test, curves with slopes like 45o show a failed test. Thus, the success of the test can be evaluated by examining the ROC curves. In a successful test, the area under the curve is expected to be great.

For comparisons between those with and without insulin resistance, the Student t−test was used in parametric groups and Mann−Whitney U test in non−parametric groups. Level of significance was accepted as p<0.05.

## RESULTS

Forty−six boys (46.2%) were evaluated as prepubertal and 81 (63.8%) as pubertal. Of the girls, 36 (25.5%) were evaluated as prepubertal and 105 (74.5%) as pubertal. In the prepubertal groups, the mean age was 8.9±1.8 years in boys and 8.3±1.4 years in girls, and the mean BMI was 28.2±5.4 kg/m^2^ in boys and 26.2±5.8 kg/m^2^ in girls. In the pubertal groups, the mean age was 13.6±1.6 years in boys and 13.2±2.0 years in girls, and the mean BMI was 30.9±4.9 kg/m^2^ in boys and 30.4±5.0 kg/m^2^ in girls. Chronological ages, BMI values, fasting blood sugar and insulin values, blood sugar and insulin values at 120 minutes, total insulin values measured during OGTT, FGIR and HOMA−IR values calculated according to gender and pubertal status are given in Tables 1 and 2.

Following OGTT, the rate of insulin resistance in the prepubertal period was 37% (n=17) in boys and 27.8% (n=10) in girls. In the pubertal children, these rates were 61.7% (n=50) in boys and 66.7% (n=70) in girls.

When the groups were separated as those with and without insulin resistance, there were no differences regarding age and BMI values, except for the girls in the pubertal period (Tables 1, 2).

We also compared the hyperinsulinemic and nonhyperinsulinemic groups for blood glucose levels at 0 and 120th minutes of OGTT. We could not find any difference in pre− and post−prandial blood glucose level in boys neither in the prepubertal nor in the pubertal groups. In prepubertal girls, we could not find any difference in fasting blood glucose level, but detected a difference in the 120−minute glucose level. In pubertal girls, there was a significant difference between 0− and 120−minute blood glucose levels between insulin resistant and nonresistant groups (Table 2).

HOMA−IR, fasting and 120−minute insulin values, FGIR and total insulin values were significantly different between the subjects of both sexes with and without insulin resistance both in the prepubertal and pubertal groups (Tables 1, 2).

HOMA−IR cut−off values for insulin resistance were calculated to be 2.67 (sensitivity 88.2%, specificity 65.5%) in boys and 2.22 (sensitivity 100%, specificity 42.3%) in girls in the prepubertal period, and 5.22 (sensitivity 56%, specificity 93.3%) in boys and 3.82 (sensitivity 77.1%, specificity 71.4%) in girls in the pubertal period.

Fasting insulin levels above 15 μU/mL in the prepubertal period, 30 μU/mL in the pubertal period and 20 μU/mL in the postpubertal period, FGIR above 6, 120−minute insulin >75

**Figures 1:**
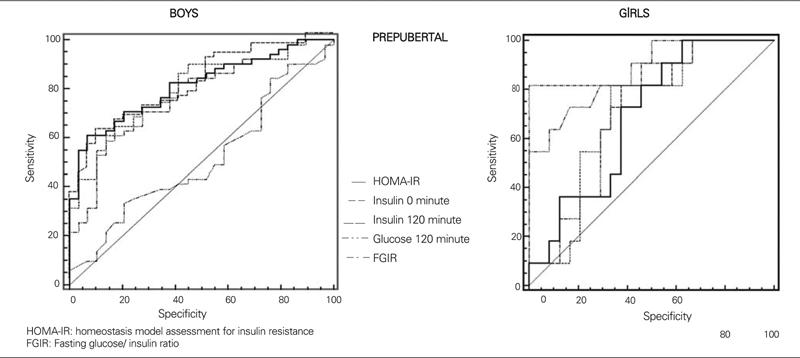
Comparison of the parameters used in determination of insulin resistance with ROC analysis in prepubertal subjects

μU/mL during OGTT and peak insulin above 150 μIU/mL are recommended as cut−off levels for hyperinsulinism and consequently as parameters showing insulin resistance (5, 28, 29). High blood sugar values at minute 120 of OGTT is an indicator of impaired glucose tolerance and therefore of insulin resistance (5). Each of these parameters (fasting insulin, insulin and blood sugar at 120^th^ minute, FGIR and HOMA−IR values) were compared using the ROC analysis regarding their importance in determination of insulin resistance according to pubertal status and gender (Figures 1, 2). With the exception of prepubertal girls, HOMAIR index was found to be the best determinant of insulin resistance in sub groups. On the other hand, insulin level at 120th minute was the best indicator of insulin resistance in the prepubertal girls. Areas under the curves in the ROC analysis of each indicator of insulin resistance according to pubertal status and gender, and 95% confidence intervals are given in Table 3.

**2:**
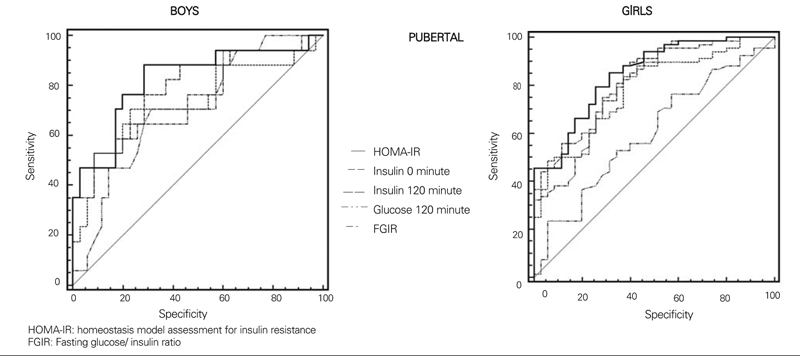
Comparison of the parameters used in determination of insulin resistance with ROC analysis in pubertal subjects

**Tables 1:**
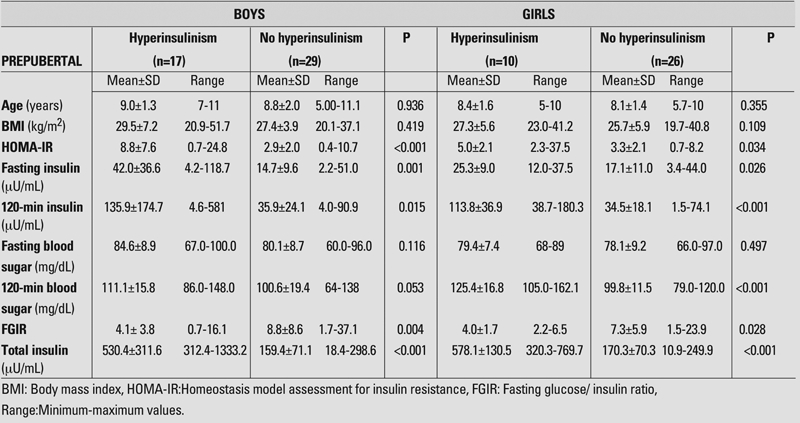
Findings in prepubertal boys and girls with and without hyperinsulinism

**2:**
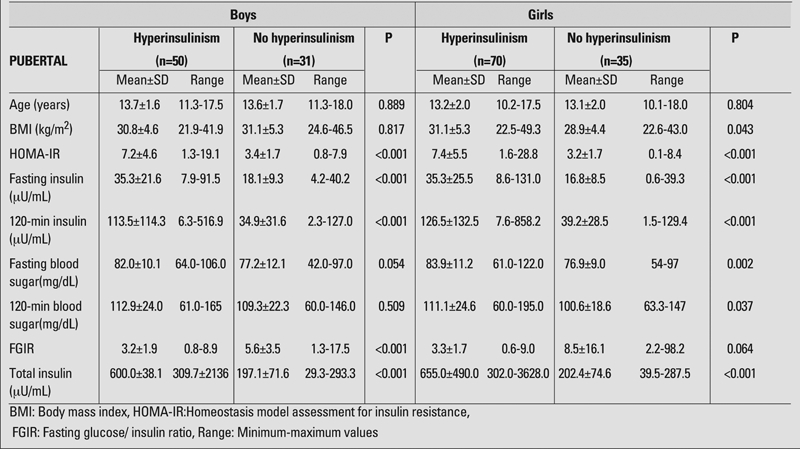
Findings in pubertal boys and girls with and without hyperinsulinism

**Table 3:**
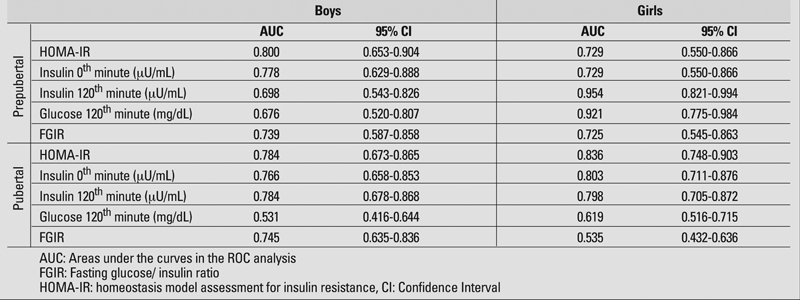
Areas under the curves in the ROC analysis of each indicator of insulin resistance according to pubertal status and gender, and 95% confidence intervals.

## DISCUSSION

Obesity is a critical risk factor for the development of insulin resistance (30, 31). Total body fat is important in the development of insulin resistance in children (30, 32). Previous studies have examined the relationship between BMI and insulin sensitivity and have shown a strong negative correlation between the values found with the euglycemic clamp test and FSIVGTT and BMI (18, 33). In our study, no difference was found in BMI values between groups with and without insulin resistance, except for pubertal girls. This finding indicates that BMI values are not always correlated with body fat ratio.

A transient insulin resistance develops in children during puberty. This insulin resistance emerging during pubertal maturation is accepted as a physiological condition rather than pathologic (32, 34). Decreased insulin sensitivity in the pubertal period causes a compensatory increase in insulin secretion (20). Cross−sectional studies have shown that insulin resistance increases with the onset of puberty, makes a peak at Tanner stage 3 and recedes to prepubertal levels at the end of puberty (15, 16, 18). Longitudinal studies have found a 30% decrease in insulin sensitivity between Tanner stages 1 and 5 (29). However, this decrease was found to return to normal at the end of puberty (35). The reasons for these changes in insulin activation and secretion are not fully understood, but are thought to be related to a mechanism enhancing the anabolic effect of insulin and growth hormone during rapid somatic growth (15, 36).

Although some studies have suggested that this change in insulin sensitivity is due to the alterations in the pubertal distribution of fat, subsequent studies have shown similar decreases in insulin sensitivity both in thin and obese children (20, 37). Furthermore, it was shown that while body fat continuously increased before and during puberty, transient insulin elevation was seen in mid puberty with a return to normal levels at the end of puberty (16). Therefore, it is not possible to explain insulin insensitivity with the change in fat distribution only.

While type 2 diabetes was previously thought to occur in elderly adults, it is now known that it also occurs in children with an increased incidence of obesity. It is claimed that adolescence is a risky period for the development of type 2 diabetes, because the transient physiological status in insulin resistance induces an extra stress on the beta cells in the pancreas (32). Therefore, adolescence is even more important for obese children regarding the risk of developing type 2 diabetes. In children of pubertal age, evaluation of insulin resistance as pathological or physiological and early intervention will help preserve the beta cell function of the pancreas. Since both obesity and puberty have a role in the development of insulin resistance, detection of increased insulin resistance of a pathological degree is of great importance, especially in obese adolescents. HOMA−IR cut−off values, which are among the most important indicators in determining insulin resistance in obese individuals, are expected to be different in prepubertal and pubertal children. In our study, when the results were compared according to the pubertal status, HOMA−IR threshold values were found to be significantly higher in the pubertal period than before puberty, in both genders. Gender has also been shown to have an effect on insulin sensitivity. Moran et al (16) reported greater insulin resistance in girls compared to boys in a large group of 357 children with the euglycemic clamp test even after adjusting for triceps and subcapsular skin thickness, BMI, waist circumference and hip circumference. Also, in a population−based study by Lee et al (38), insulin resistance was found to be greater in girls than in boys after adjusting for weight, race and race/ethnicity.

In our study, while no difference for gender was detected in HOMA−IR cut−off values in the prepubertal period, cut−off values in the pubertal period were higher in boys than in girls. Because insulin resistance is greater in girls, HOMA−IR cut−off values are expected to be lower in boys. We do not have an explanation for this gender differences. However, it is important that, in the evaluation of insulin resistance in the pubertal period, different threshold values should be used for boys and girls.

Moran et al (16) reported that gender differences for insulin resistance disappeared among children with BMI values greater than 27 kg/m^2^ and therefore, gender difference can become insignificant in morbid obesity. In our study, no difference between genders was observed despite the mean BMI values around 30 kg/m^2^ in all children.

Due to the fact that our sample size was small , we are not able to propose precise cut−off limits based on the results of this study. However, based on this experience, we believe that determination of fasting glucose and insulin levels in epidemiological studies will provide much more accurate results with which we can calculate HOMA−IR.

Another weak point of this study is that we have not performed a clamp study, which is the gold standard. Thirdly, we have taken the same criteria for insulin resistance in pre−and pubertal children on OGTT. However, there are noestablished separate values for these periods.

In conclusion, obesity and puberty are important factors for the development of insulin resistance. Both gender and pubertal status should be considered when evaluating insulin resistance in obese children, and risk groups should be identified accordingly. Gender differences should also be considered in the evaluation of insulin resistance. We recommend that HOMA−IR cut−off values, which are among the most widely used indicators of insulin resistance, be used in this evaluation, also taking into account both pubertal status and gender differences. Thus, particularly in a period critical for the development of type 2 diabetes such as the pubertal period, risk groups can be determined in a more reliable manner, and measures can be taken.
